# Population demography of Oldham’s leaf turtle (*Cyclemys oldhamii*) in protected and disturbed habitats in Thailand

**DOI:** 10.7717/peerj.7196

**Published:** 2019-07-02

**Authors:** Sengvilay Seateun, Nancy E. Karraker, Bryan L. Stuart, Anchalee Aowphol

**Affiliations:** 1 Department of Zoology, Faculty of Science, Kasetsart University, Bangkok, Thailand; 2 Department of Natural Resources Science, University of Rhode Island, Kingston, RI, United States of America; 3 North Carolina Museum of Natural Sciences, Raleigh, NC, United States of America

**Keywords:** *Cyclemys*, Demography, Disturbed habitat, Morphometrics, Population size, Sexual dimorphism, Survival rate, Thailand, Turtles

## Abstract

**Background:**

Freshwater turtle populations are vulnerable to a range of human activities because of particular life history attributes, and anthropogenic impacts can cause shifts in demographic traits, including survival, density and population structure. Asian freshwater turtles have undergone dramatic population declines in recent decades principally because of collection for food, pet, and traditional medicine markets. Despite this, few studies have been conducted on the population demography of these turtles, thereby limiting our understanding of population trends and the development of conservation actions. Oldham’s leaf turtle (*Cyclemys oldhamii*) is one of the most commonly traded turtles in Asian markets, but previous published studies have focused solely on systematics.

**Methods:**

We conducted a mark-recapture study of *C. oldhamii* at three sites in northeastern Thailand—a protected stream, a degraded stream, and human-constructed ponds—and evaluated differences in survival, density, population structure, and sexual dimorphism among sites.

**Results:**

We captured 77 turtles at the protected stream, 67 at the constructed ponds, and two in the degraded stream. Survival was 12% lower and density was 35% lower in the constructed ponds than in the protected stream. Size class structure was skewed toward smaller individuals at the constructed ponds, and both sites exhibited subadult-skewed age class structure. Sex ratios were not statistically different than 1:1 at either site and did not differ between sites. We did not document sexual dimorphism in either population.

**Discussion:**

Explanations for lower survival, lower densities, and skewed size class structure at the constructed ponds include collection for consumption or Buddhist prayer release locally, collection for illegal export from Thailand, predation by domestic dogs associated with humans living nearby, or lower habitat quality. Evidence from our study suggests that collection, either for local use or export, is the most likely explanation for differences in demographic characteristics between the two sites. The information gained from this study may contribute to a status assessment for *C. oldhamii* and development of conservation actions should they become necessary to protect populations in Thailand.

## Introduction

Turtles exhibit life history traits that make their populations particularly vulnerable to human-caused impacts in their environments (Heppell, 1998), as compared with some other vertebrate groups. Freshwater turtle species, in particular, are generally characterized as having delayed reproduction, with sexual maturity not occurring until 7–14 years (e.g., Congdon, Dunham & Van Loben Sels, 1993; Frazer, Gibbons & Greene, 1990), low subadult survivorship (e.g., Shine & Iverson, 1995), and low fecundity (e.g., Iverson, 1991; Sung, Hau & Karraker, 2014), and in some species, individuals may not breed annually (Litzgus & Mousseau, 2006). These traits make populations less resilient to anthropogenic impacts and can lead populations into an extinction vortex (e.g., Shoemaker et al., 2013) from which many are unlikely to recover.

Freshwater turtle populations have been demonstrated to be vulnerable to a range of human activities. Population declines have been associated with actions seemingly as benign as opening up protected areas for hikers (Garber & Burger, 1995), more invasive activities such as pollution and sedimentation of aquatic habitats (Dodd, 1990), predation by non-native predators (Fordham, Georges & Brook, 2007), and direct removal of individuals for trade (Lyons, Natusch & Shepherd, 2013). In addition to obvious changes such as decreasing abundances in carefully monitored populations which may indicate populations compromised by anthropogenic impacts, other demographic traits may signal human-induced changes in populations that could lead to declines over time. The demographic or ecological traits potentially shifted by humans’ activities depends on the life history of the focal species and the particular type of human-caused perturbation. For example, expansion of non-native plants and decreases in habitat suitability have been associated with a decline in survival rate in a turtle species with unique habitat requirements (Sirois et al., 2014). Conversion of mature forest to early-successional habitat around wetlands was associated with an increase in the home range size of a turtle species that spends about 30% of time on land (Buchanan, Buffum & Karraker, 2017). Skewed size class and age class structures were observed in populations of a highly aquatic species targeted by illegal collectors (Sung, Karraker & Hau, 2013). Populations impacted by activities of humans may exhibit reduced abundances but other indicators may be evident in their demography, and this may be especially true for species whose populations are exposed to harvesting.

Asian turtle populations have been inordinately impacted by harvesting to supply markets for food, traditional Chinese medicine, and the international pet trade (Auliya et al., 2016; Kharel & Chhetry, 2012; Stuart & Platt, 2004; Van Dijk, Stuart & Rhodin, 2000) in a critical conservation issue known as the ‘Asian turtle crisis.’ In conservation assessments completed by the International Union for the Conservation of Nature (IUCN), 80% of Asian turtles appear in threatened categories with more than 50% of those as Endangered or Critically Endangered (IUCN, 2018). For most species, there has been almost no research beyond the initial species description and subsequent efforts to gain further taxonomic resolution on related species. In fact, populations of many Asian turtle species have declined to such low levels that it has become difficult to study their ecology (Shen, Pike & Du, 2010) or population biology, understandings of which are necessary for development of conservation action plans. Quantifying the demographic structure of turtle populations is integral for understanding population trends and conservation needs (Bury, Germano & Bury, 2010), particularly in light of rapid land use change in some parts of Southeast Asia (Sodhi et al., 2004).

Despite robust populations remaining in some parts of Southeast Asia, as one of the top five turtle taxa sold in Hong Kong and mainland China markets (Cheung & Dudgeon, 2006), Asian leaf turtles (*Cyclemys* spp.) are of conservation concern. We examined three populations of Oldham’s leaf turtle, one in a natural protected area and two in disturbed areas, to quantify the effects of humans’ activities on survival, density, age class structure, size class structure, sex ratio, and degree of sexual dimorphism. Based on documented vulnerabilities of freshwater turtles to anthropogenic disturbance, we developed and tested a set of hypotheses. We hypothesized that the populations in the two disturbed habitats would exhibit lower survival and lower densities than the protected stream because of degraded habitats and increased risk of illegal collection. We expected that the degraded stream and human-constructed ponds would have a subadult-skewed age class structure because adult numbers would be reduced by collection. In contrast, the protected stream should exhibit an adult-skewed age class structure. Finally, we hypothesized that we would document male-skewed sex ratios in the two disturbed habitats because females are often targeted or are more easily found by collectors. In contrast, the protected population was predicted to have an even sex ratio. We expected all three populations to exhibit sexual dimorphism, with females being slightly larger than males since female-biased sexual dimorphism has been reported in many aquatic turtles, including the family Geoemydidae such as *Malayemys macrocephala* from Thailand (Brophy, 2006).

## Materials and Methods

### Study species and sites

Oldham’s leaf turtle (*Cyclemys oldhamii*) is distributed in Cambodia, Laos, Myanmar, Thailand, and Vietnam (Rhodin et al., 2017). The species is principally associated with lower-elevation aquatic habitats generally surrounded by forest (Rai, 2004; Stuart et al., 2010) and is known to move between aquatic and terrestrial habitats (Durkin, 2012). Turtle species in the genus *Cyclemys* are currently placed in the Red List Category Near Threatened by IUCN (2018), and in 2013, all *Cyclemys* species were upgraded to Appendix II by the Convention on International Trade in Endangered Species (CITES, 2016), which places restrictions on trade of these species between CITES signatory countries. Although *Cyclemys* spp. appear frequently in trade in several countries (Cheung & Dudgeon, 2006; Stuart & Platt, 2004; Vamberger et al., 2017), the population demography and ecology of turtles in this genus are largely unstudied.

We conducted this research within the Indo-Burma biodiversity hotspot, a geographic expanse that contains the highest proportion of the world’s turtle diversity (Buhlmann et al., 2009). We focused on three study sites in and around Sakaerat Biosphere Reserve, a United Nations-designated reserve located in Nakhon Ratchasima Province in northeastern Thailand. The first study site, Sakaerat Environmental Research Station (SE; N14 30.344; E101 55.135), is a protected habitat that contains a nearly pristine, natural stream system characterized by a moderate gradient and dominated by bedrock and boulder substrates. Mean width of the stream channel is 3.8 m. During the rainy season, many pools can be found along the streams at SE, but during the dry season only a few of these pools contain water and the stream channel is largely dry. The primary forest surrounding the stream is structurally complex, mature, evergreen, secondary forest with legacy trees up to 400 years old. This forest type is dominated by trees in the family Dipterocarpaceae and is generally characterized by a dense shrub and liana layer and a closed canopy. Collection of food or other resources by villages living nearby is prohibited in this area.

The second study site, Wang Nam Khiao Forestry Student Training Station of Kasetsart University (KU; N14 29.997; E101 56.442), is a highly disturbed natural stream that runs parallel to a highway and is flanked by small villages. The stream is low gradient and dominated by cobble-sized and smaller substrates, including large amounts of sediment, possibly contributed by nearby construction of a small check dam. Mean stream width is 5.0 m and the stream flows year-round. The stream is highly turbid, exhibits signs of periodic algal blooms, and contains some garbage. Sparse riparian vegetation is present. People living nearby collect water from the stream for use in their houses and for crops and livestock. Other resources, such as fish and crabs, are extracted from the stream, and plants are collected from the surrounding area.

The third study site, Sakaerat Silvicultural Research Station (SS; N14 30.339; E101 54.131), contains four human-constructed ponds (two measuring about 75 × 60 m in size and two measuring 17 × 10 m in size), constructed to provide a water source for nearby forestry plantations. The two larger ponds are located 9.5 m apart and contain water year-round. The two smaller ponds were located 716 m from the larger ponds. The smaller ponds dry annually, and as they are located immediately adjacent to each other and separated by only a small berm, they were treated as one pond for the purposes of this study. Trees of *Acacia* sp., *Leucaena* sp., and *Eucalyptus* sp. are grown in the plantation area surrounding these ponds; the *Acacia* sp. and *Leucaena* sp. are about 30 years old and the *Eucalyptus* sp. are about 15 years old. Plantation workers live near the artificial ponds and domestic dogs are present in the area.

The three study sites (SE, KU, and SS) are located >3 km from each other, with barriers such as roads and deforested areas in between, so we considered them to be separate populations. Elevation across the study areas ranges from 250−762 m. The climate is tropical with a mean annual air temperature of 26.4 °C, mean annual maximum temperature of 35 °C, and mean annual low temperature of 16 °C. The wet season spans from May to mid-October, with rainfall peaks in May and September and mean annual rainfall of 1025 mm the dry season, which occurs from November to April. The environment of Sakaerat Biosphere Reserve has been described in detail by Inger & Colwell (1977).

### Animal sampling

We used funnel traps baited with canned fish to capture turtles. Each trap contained a plastic bait tube into which holes were drilled so that turtles could smell the bait but not eat it and a plastic bottle that served as a flotation device to ensure turtles’ access to the surface to breathe. Trapping was conducted along a 1.8-km section of a protected natural stream at SE, with an average stream width of 3.8 m, for a total area of approximately 0.68 ha. We trapped a 1.3-km section of a disturbed stream at KU totaling approximately 0.65 ha. Human-constructed ponds that we trapped at SS made up a total area of approximately 0.93 ha. All study areas were trapped three days and two nights per month. Sites SE and SS were trapped monthly from July 2014 to December 2016, for a total of 20 months at each site because of periodic drying of some habitats. Site KU was trapped monthly from September 2014–January 2015 and March–December 2015, for a total of 12 months. One trap was set approximately every 30 m along the lengths of the streams or around the perimeter of the ponds so that trapping effort was comparable among habitat types. Traps were set in late afternoon and checked the following morning. We permanently marked each subadult and adult turtle by notching a unique series of marginal scutes with a triangular metal file (Cagle, 1939), so that individual turtles could be identified. Hatchlings were not marked because their shells were still soft and marking at that stage can result in permanent shell damage as the turtle grows. Thus, hatchlings could not be individually identified and were not included in analyses. For each turtle captured, we measured the following morphological characters: straight-line carapace length (CL), carapace width (CW), shell height (SH), plastron length (PL), and plastron width (PW) to the nearest 0.1 mm using calipers. Body mass was measured with a spring scale to the nearest 1 g for turtles <100 g, to the nearest 10 g for turtles >100 g, and to the nearest 20 g for turtles >1,000 g. We determined sex of turtles using external secondary sexual characteristics. As in many other freshwater turtles (Rai, 2004; Sung, Karraker & Hau, 2013), males have a longer pre-cloacal tail length and thicker tail base than do females. All turtles not exhibiting sexually-dimorphic characters, and thus not yet sexually mature, were considered to be subadults.

As Sakaerat Biosphere Reserve has a distinct annual warm, wet season alternating with a cooler, dry season, lines of arrested growth form on shell scutes of turtles on an annual basis. For each turtle, we counted these annuli on at least two scutes on both the carapace and plastron. When freshwater turtles reach about 12–15 years old, the annuli begin to overlap each other and thus older turtles cannot be aged reliably. In addition, the carapace or plastron may become worn and smooth over many years, leading to inaccuracy in growth estimation by growth ring counts (Litzgus & Brooks, 1998). We found that we could reliably count growth rings up to 15 years of age for *C. oldhamii*. Turtles for which we observed overlap in growth rings or for which the scutes were worn and smooth, we classified age as >15 years. After measurements, turtles were released at the point of capture.

### Population structure, survival, and density

Trapping at KU was discontinued after 12 months, due to extremely low capture rates, and population analyses were not conducted for this site. As sites SE and SS were trapped for 20 months, we collapsed these trapping sessions into five, four-month survey bouts for analysis. We used Cormack–Jolly–Seber models to estimate annual survival and recapture probabilities separately for each site, including the group attributes age/sex class (subadult, male, female) and site (SE, SS). We also compared size class structure between the two populations. The timing of the wet and dry seasons varied widely among years so we were not able to evaluate the importance of season to survival or recapture probability. We constructed models that evaluated group attributes in the context of constant and time-dependent survival and recapture probabilities. We evaluated model fit to the data with a bootstrapping goodness of fit test of the most general model that included time-dependent survival and time-dependent recapture probability. We estimated population size for each site separately using open population Jolly Seber models and evaluated candidate models with constant and time-dependent survival and recapture probabilities and time-dependent probability of entry. Using mean population size estimates from the top model for each site, we generated density estimates for each population. Population analyses were conducted in Program Mark (White, Colorado State University, USA. Available online at: http://www.phidot.org/software/mark/index.html). Numbers of males and females at each site and sex ratios between sites were compared using chi-square tests with the R program (R Development Core Team, 2014). We determined percentage of individuals within each age/sex class recaptured of the total captured within each class. We compared proportion of subadults between sites using a chi-square test.

### Sexual dimorphism

Distributions of morphometric data were tested for normality using a Shapiro–Wilk’s test. Data for all morphological parameters for both sexes were normally distributed, except for shell height and plastron length in females, which deviated slightly from normal, so we used parametric tests to examine sexual dimorphism. We quantified morphological differences between sex and site and an interaction term (sex × site) and compared them using two-way ANOVA. Analyses were conducted using the statistical software SPSS and differences were considered significant at *α* = 0.05.

Scientific research permits were provided by the Sakaerat Silvicultural Research Station, Royal Forestry Department (No.1607.5/2201), Sakaerat Environmental Research Station (No. 5201/106), Faculty of Forestry, Kasetsart University (No. 0513.10601/3358) and National Research Council of Thailand (No. 0002/8188). This research was approved by the Institutional Animal Care and Use Committee, Kasetsart University (Permit # ACKU60-SCI-003).

**Figure 1:**
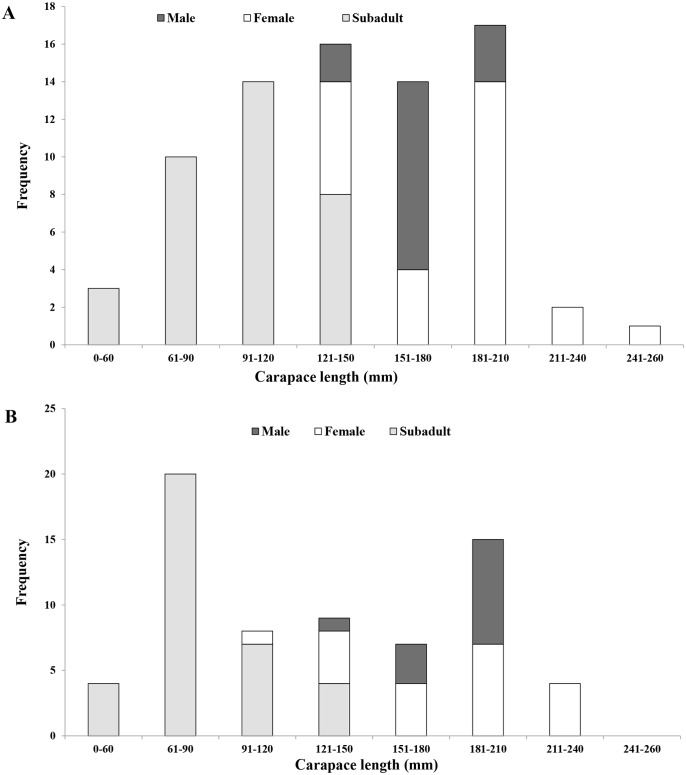
Carapace lengths of Oldham’s leaf turtle (*Cyclemys oldhamii*). Frequency distribution of carapace lengths turtles captured at (A) Sakaerat Environmental Research Station (SE) and (B) Sakaerat Silvicultural Research Station (SS) in Sakaerat Biosphere Reserve, Nakhon Ratchasima, Thailand from July 2014 to December 2016.

## Results

We conducted 20 surveys each on the protected stream at SE and the human-constructed ponds at SS and 12 surveys of the disturbed stream at KU. We captured a total of 146 turtles, including 77 at SE, 67 at SS, and 2 at KU. We made no observations of turtles moving between the three sites during the study period.

### Population structure

**Figure 2:**
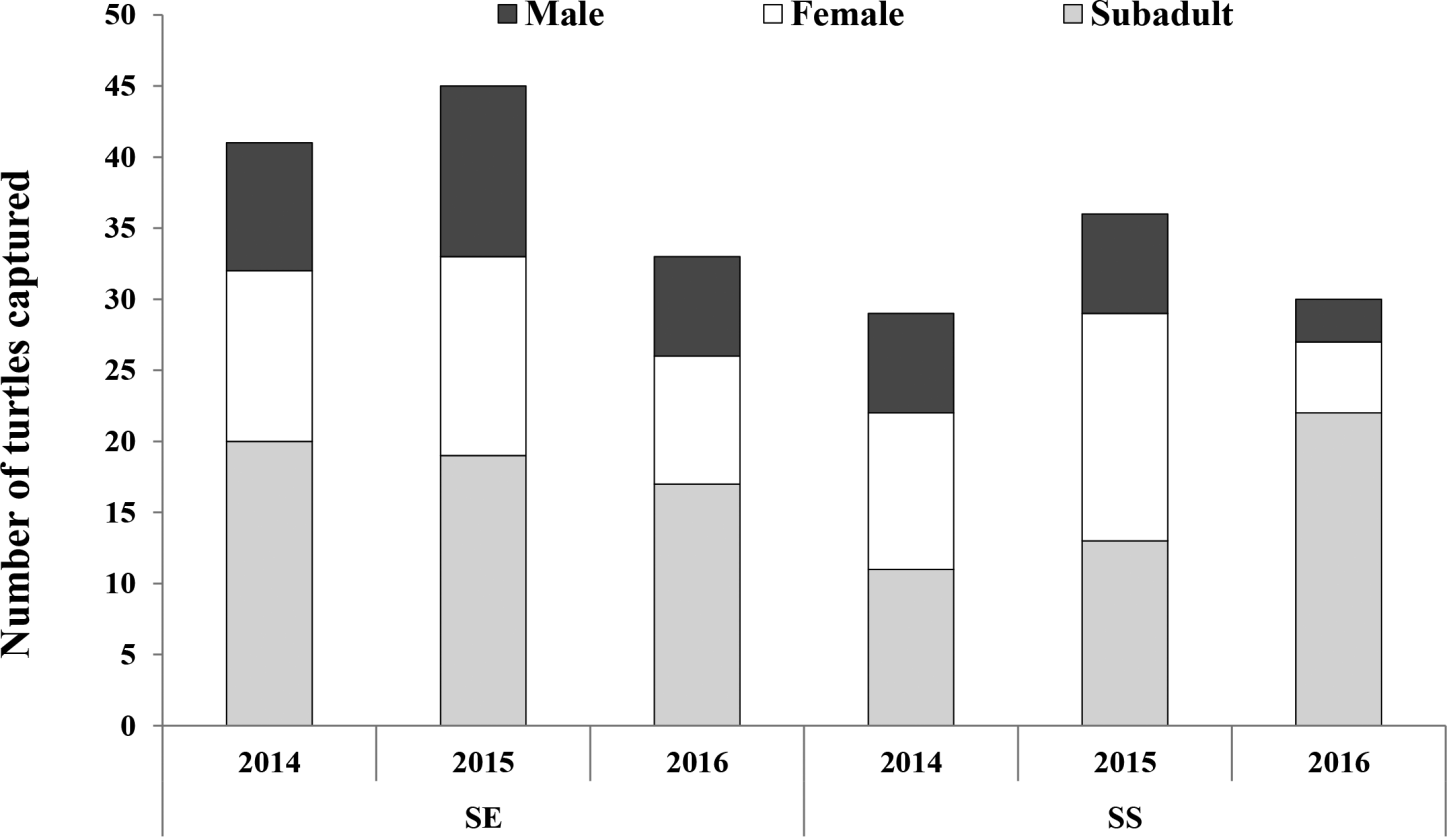
Population structure of Oldham’s leaf turtle (*Cyclemys oldhamii*). Population structure by year of turtles captured at Sakaerat Environmental Research Station (SE) and Sakaerat Silvicultural Research Station (SS) study sites in Sakaerat Biosphere Reserve, Nakhon Ratchasima, Thailand from July 2014 to December 2016.

At SE, where we sampled a protected, relatively pristine stream, a total of 77 individuals were captured and marked during the study period, consisting of 15 (19%) males, 27 (35%) females and 35 (45%) subadults. The sex ratio (male: female) was slightly female-skewed but numbers of males and females were not significantly different (1:1.8; *X*^2^ = 3.426, *P* = 0.064). The most frequent size class represented was adults with carapace length ranging from 180–210 mm (Fig. 1A), making up 22% of all captures. Forty-five individuals were recaptured at least once, representing 58% of all turtles marked (Fig. 2). Of the 77 individuals captured at SE, we recaptured 73% of marked males, 41% of marked females, and 66% of marked subadults.

At SS, which was made up of three constructed ponds that received frequent human-caused disturbance, we captured and marked 67 individuals, including 12 (18%) males, 20 (30%) females, and 35 (52%) subadults. Subadults in the size class 61–90 mm were the most frequent size class captured, constituting 30% of all captures (Fig. 1B), and individuals with carapace length >240 mm were absent from the population. Sex ratio was slightly female-skewed, but numbers of males and females did not differ statistically (1:1.7; *X*^2^ = 2.00, *P* = 0.157). We recaptured 61% of all individuals, including 66% of marked males, 60% of marked females, and 60% of marked subadults.

At KU, which consisted of a degraded stream adjacent to a village, we captured only one female and one subadult between September 2014 and September 2015. To maximize effort at other sites, we discontinued surveys at KU and were unable to include data from that site in the population analysis.

Sex ratio (*X*^2^ = 0.01, *P* = 0.931) and proportion of subadults (*X*^2^ = 0.72, *P* = 0.395) did not differ statistically between SE and SS.

### Population survival and density

Site strongly influenced survival rates. Survival estimates for all age/sex classes combined were 12% higher (Table 1) at the protected stream at SE than at the constructed ponds at SS. Similarly, survival estimates were higher for subadult, male, and female turtles at SE than at SS (Table 1). Confidence intervals on survival estimates for males and females at both sites were relatively broad, so estimates for these age/sex classes should be considered cautiously. A bootstrapping goodness of fit test indicated good fit (c-hat = 1.27) of the most general model to the data.

**Table 1:** Model estimates of apparent survival (*ψ*) of Oldham’s leaf turtle (*Cyclemys oldhamii*). *Cyclemys oldhamii* in a protected stream at Sakaerat Environmental Research Station (SE) and human-constructed ponds at Sakaerat Silvicultural Research Station (SS) in Sakaerat Biosphere Reserve, Nakhon Ratchasima, Thailand from 2014–2016.

Population	Sex/age class	*φ*	SE	95% CI	Model
SE	overall	0.815	0.062	0.664−0.907	phi(site)p(.)
	subadult	0.769	0.077	0.587−0.886	phi(age/sex*site)p(.)
	male	0.949	0.093	0.300−0.999	phi(age/sex*site)p(.)
	female	0.683	0.157	0.343−0.899	phi(age/sex*site)p(.)
					
SS	overall	0.694	0.057	0.573−0.793	phi(site)p(.)
	subadult	0.740	0.085	0.545−0.872	phi(age/sex*site)p(.)
	male	0.862	0.222	0.140−0.996	phi(age/sex*site)p(.)
	female	0.614	0.086	0.439−0.764	phi(age/sex*site)p(.)
					
Both sites	overall	0.751	0.042	0.660−0.824	phi(group)p(.)
	subadult	0.756	0.057	0.629	Models averaged
	male	0.937	0.091	0.420	Models averaged
	female	0.631	0.073	0.480	Models averaged

For the aquatic portion of the habitats used by *C. oldhamii*, we estimated population densities of 57.4 turtles per ha in the protected stream and 37.7 turtles per ha in the constructed ponds.

### Sexual dimorphism

We found no statistically significant differences between body size parameters for males and females at either site, and no evidence of differences in sexual dimorphism between the protected and disturbed site (Table 2). *P*-values for parameters sex, site, and the interaction between sex and site were ≥0.099 for carapace length, carapace width, plastron length, plastron width, shell height, and mass.

**Table 2:** Morphological characters of male, female, and subadult Oldhams leaf turtle (*Cyclemys oldhamii*). *Cyclemys oldhamii* captured at Sakaerat Environmental Research Station (SE) and Sakaerat Silvicultural Research Station (SS) study sites in Sakaerat Biosphere Reserve, Nakhon Ratchasima, Thailand from July 2014 to December 2016. n refers to total number of individuals captured at each site over the entire survey period.

	Male		Female		Subadult	
Morphologicalcharacter	Mean (SD)	Range	Mean (SD)	Range	Mean (SD)	Range
*Sakaerat Environmental Research Station (SE)*						
n	15	–	27	–	35	–
Carapace length	167 (16)	139−193	185 (31)	134−260	97 (26)	57−143
Carapace width	130 (10)	112−146	138 (16)	108−177	86 (21)	53−120
Shell height	57 (5)	50−65	62 (11)	45−87	34 (9)	20−52
Plastron length	153 (14)	130−174	169 (28)	123−228	88 (25)	51−134
Plastron width	100 (8)	87−116	108 (14)	82−138	63 (17)	36−91
Mass	574 (150)	312−834	775 (346)	302−1757	146 (102)	23−335
*Sakaerat Silvicultural Research Station (SS)*						
n	12	–	20	–	35	–
Carapace length	183 (18)	144−209	179 (37)	117−232	79 (26)	49−138
Carapace width	138 (10)	123−156	138 (20)	98−163	71 (21)	46−120
Shell height	63 (7)	50−78	65 (14)	41−80	29 (9)	18−47
Plastron length	164 (17)	130−188	165 (35)	102−213	70 (24)	41−127
Plastron width	104 (8)	93−117	107 (19)	72−145	52 (17)	31−94
Mass	757 (226)	380−1200	806 (395)	198−1500	89 (92)	16−347

## Discussion

### Population structure

Populations of *C. oldhamii* in both the protected stream and the human-created ponds exhibited even sex ratios, or ratios not statistically different from one male to one female. Even sex ratios are commonly reported in populations of aquatic turtles (Bury, Germano & Bury, 2010; Gibbons & Lovich, 1990). Skewed sex ratios are often related to seasonal behavioral differences and differential mortality between sexes, temperature-dependent sex determination, or differences in emigration, immigration and habitat use between sexes (Bull, 1985; Gibbons & Lovich, 1990; Smith & Iverson, 2002). Although we have an understanding of the types of environmental and anthropogenic drivers potentially associated with shifts in sex ratios, the mechanisms responsible in individual populations are often difficult to identify. Male-skewed sex ratios have been documented in populations of a number of turtle species (e.g., Reeves & Litzgus, 2008; Smith & Iverson, 2002), and in some cases these skewed sex ratios have been associated with declining populations (e.g., Hall, Henry & Bunck, 1999). Our study of *C. oldhamii* provides two years of baseline data on sex ratios at the two sites and results suggest that the sex ratio is even and that differential mortality of females has not occurred at our study sites recently. Longer-term monitoring is needed to assess population trends.

We documented similar age class structure between the two study sites, with subadults making up 45% of the sampled population at the protected stream and 52% at the human-created ponds. Although a higher proportion of subadults in a population could indicate increased recruitment and potentially a healthy population (Seburn, 2003), it may also signal reduced numbers of adults in the population due to collection, as has been demonstrated in other freshwater turtle species (e.g., Sung, Karraker & Hau, 2013). An alternative explanation may be related to differences in habitat use between adult and subadult turtles. Adult *C. oldhamii* leave aquatic habitats occasionally to feed in the forest. Subadults are more prone to predation on land and, as with most aquatic turtles, probably rarely leave water. Thus, subadult *C. oldhamii* may be sampled more frequently than adults, when trapping occurs only in their aquatic habitats, as in the case of our study. Assessments of age class structure should take into consideration the different life histories of species, such as *C. oldhamii*, that exhibit phenological shifts in habitat use.

Notably, the most frequent size class captured at the two sites differed. Adult turtles measuring 181−210 mm in carapace length were the most common size class captured in the protected stream, and subadults measuring 61−90 mm were most common in the constructed ponds. In addition, turtles of the largest size class (>240 mm) were absent from the constructed ponds, but only one individual represented this size class in the protected stream. Skewed size classes or the absence of larger size classes in turtle populations can be an indicator of collection by humans (Sung, Karraker & Hau, 2013), because larger turtles may be more easily spotted in their habitats or because they may garner a higher price. This can impact a population as the largest females, which generally make the largest reproductive contributions, would be removed from the population. The population at the constructed ponds are likely impacted by illegal collection as evidenced by the difference in size class distributions between the two sites, the absence of the largest turtles, and the proximity of the constructed ponds to humans.

### Population survival and density

Average survival was 12% lower at the constructed ponds than at the protected stream, and lower survival was consistent among age/sex classes at the constructed ponds. Turtle densities were nearly 35% lower in the constructed pond population than in the protected stream population. Possible explanations for lower survival and densities in the disturbed pond habitat may include a combination of illegal harvesting of turtles, predation by domestic dogs, or lower habitat quality. Due to the scale and scope of the turtle trade, we must consider the possibility that illegal harvesting contributes to the lower survival and density of turtles from the constructed ponds. To our knowledge, turtles are not consumed by people in northeastern Thailand, but they may be collected from the wild and sold in markets to be freed as a part of Buddhist prayer release rituals or for illegal export. In Buddhism, release of any animal is believed to bring good merit to the releaser (Severinghaus & Chi, 1999), and the release of turtles is believed to bring the emancipator a long life. Nature reserve staff were not aware of any collection of *C. oldhamii* within the reserve, and weekly visits to the nearest market in Pak Tong Chai in August and September 2010 and 2011 yielded only lowland wetland-dwelling snail-eating turtles (*Malayemys macrocephala*). Although we do not have any direct evidence of harvesting of turtles, it is possible that it occurs on a limited basis.

An alternative explanation for lower survival and density at the constructed ponds is that domestic dogs living around humans nearby are preying on turtles. However, we consider this explanation unlikely. Dogs are probably able to feed only on the youngest subadult turtles, because the rigid shells of older subadults and adults would preclude predation by a dog. In addition, subadults of size 61–90 mm were the most frequently captured individuals at the constructed ponds.

The constructed ponds may provide lower quality habitat or fewer resources potentially leading to lower survival or density of turtles. Although this may be a contributing factor, we also do not find strong support for this explanation. The two larger constructed ponds contained water year-round, thus potentially allowing turtles to remain active and feed during the cooler, drier season. We cannot entirely rule out differences in resources contributing to lower survival and density but consider it unlikely.

Importantly, density estimates for our two populations pertain only to their aquatic habitats and are likely systematic overestimates, for both populations, with regard to densities of *C. oldhamii* across the broader landscape of aquatic and terrestrial habitats used by the species. We urge caution in the use of these estimates and emphasize that they serve most appropriately for comparing densities of *C. oldhamii* in their aquatic habitats only. Future efforts should incorporate information on use of terrestrial habitats by these turtles to generate more precise estimates of density across both aquatic and terrestrial habitats (Ivan, White & Shenk, 2013).

### Sexual dimorphism

Counter to our prediction, we did not observe sexual dimorphism in *C. oldhamii*. Sexual dimorphism has been documented in many other freshwater turtles, including the snail-eating turtle (*Malayemys macrocephala*) in Thailand (Brophy, 2006), and is attributable to a larger reservoir for eggs in females or the need for size in males that engage in combat (Gibbons & Lovich, 1990). Although we did not document sexual dimorphism, reductions in numbers of turtles in larger size classes at the disturbed site may have influenced this result. Larger sample sizes in unimpacted habitats may be needed to gain further resolution on sexual dimorphism in *C. oldhamii.*

## Conclusions

To our knowledge, this is the first study to be conducted on the population demography of *Cyclemys oldhamii*, a species that is common in the global turtle trade. *Cyclemys oldhamii* is recognized by CITES (2019) as an Appendix II species, which controls trade to avoid impacts to populations. Thailand does not currently have a CITES-approved export quota, indicating that *C. oldhamii* cannot be legally exported from the country. Information on illegal export of this species from Thailand is not available, but is likely occurring given the frequency of this turtle in international trade (Cheung & Dudgeon, 2006). Although recognized as a distinct species from *C. dentata*, a status assessment has not been completed for *C. oldhamii* by the International Union for the Conservation of Nature because of unresolved issues with taxonomy and distribution (Asian Turtle Trade Working Group, 2000). We hope that the information gained from this study will aid in the undertaking of a status assessment for *C. oldhamii* and provide information that may be necessary for any future consideration of export quotas of *C. oldhamii* from Thailand.

Three lines of evidence—lower survival, lower density, and skewed size classes—at the constructed ponds suggest that collection may be occurring at that site. We cannot exclude either illegal harvesting for export or Buddhist prayer release as the mechanism that may be driving collection. Recently, the Thai Buddhist Council announced a ban on bird trade associated with ceremonial release (IUCN, 2017), but such protections have not been made for turtles. Notably, Buddhist ceremonies impact native turtle populations by the capture of native turtles for release to different locations, and also by the purchase and release of invasive red-eared sliders (*Trachemys scripta*). This turtle from the Americas has been introduced to the Republic of Korea, Taiwan, Vietnam (Ramsay et al., 2007), and Thailand, where it is sold as a pet, and is suspected to outcompete native Asian turtles. We recommend that the Thai Buddhist Council invoke a similar resolution for turtles as they have for birds and that the Thailand Government support such an effort. In addition, we urge the Thailand Government to take steps to ensure that illegal collection of turtles from national parks and reserves is minimized. As turtle populations become depleted in other Southeast Asian countries because of legal and illegal export and domestic uses, pressure will be increasingly exerted on turtle populations in countries like Thailand to meet the demands of an insatiable global trade.

##  Supplemental Information

10.7717/peerj.7196/supp-1 Data S1 Raw data of *Cyclemys oldhamii* at Sakaerat Environmental Research Station (SE)

10.7717/peerj.7196/supp-2 Data S2 Raw data of *Cyclemys oldhamii* at Sakaerat Silvicultural Research Station (SS)

## Additional Information and Declarations

### Field Study Permissions

The following information was supplied relating to field study approvals (i.e., approving body and any reference numbers): Scientific research permits were provided by the Sakaerat Silvicultural Research Station, Royal Forestry Department (No.1607.5/2201), Sakaerat Environmental Research Station (No. 5201/106), Faculty of Forestry, Kasetsart University (No. 0513.10601/3358) and National Research Council of Thailand (No. 0002/8188).

### Data Availability

The following information was supplied regarding data availability: The raw data are available as Supplemental Files.

## References

[ref-1] Asian Turtle Trade Working Group (2000). *Cyclemys dentata* (errata version published in 2016). The IUCN Red List of Threatened Species 2000.

[ref-2] Auliya M, Altherr S, Ariano-Sanchez D, Baard EH, Brown C, Brown RM, Cantu J-C, Gentile G, Gildenhuys P, Henningheim E (2016). Trade in live reptiles, its impact on wild populations, and the role of the European market. Biological Conservation.

[ref-3] Brophy TR (2006). Allometry and sexual dimorphism in the snail-eating turtle *Malayemys macrocephala* from the Chao Phraya River Basin of Central Thailand. Chelonian Conservation and Biology.

[ref-4] Buchanan S, Buffum B, Karraker N (2017). Responses of a spotted turtle population (*Clemmys guttata*) to creation of early-successional habitat. Herpetological Conservation and Biology.

[ref-5] Buhlmann KA, Akre TS, Iverson JB, Karapatakis D, Mittermeier RA, Georges A, Rhodin AG, Van Dijk PP, Gibbons JW (2009). A global analysis of tortoise and freshwater turtle distributions with identification of priority conservation areas. Chelonian Conservation and Biology.

[ref-6] Bull J (1985). Sex ratio and nest temperature in turtles: comparing field and laboratory data. Ecology.

[ref-7] Bury RB, Germano DJ, Bury GW (2010). Population structure and growth of the turtle *Actinemys marmorata* from the Klamath–Siskiyou ecoregion: age, not size, matters. Copeia.

[ref-8] Cagle FR (1939). A system of marking turtles for future identification. Copeia.

[ref-9] Cheung SM, Dudgeon D (2006). Quantifying the Asian turtle crisis: market surveys in southern China, 2000–2003. Aquatic Conservation: Marine and Freshwater Ecosystems.

[ref-10] CITES (2016). UNEP-WCMC Species Database: CITES-Listed Species. Convention on the International Trade in Endangered Species. https://cites.org/eng/node/7691.

[ref-11] CITES (2019). Cyclemys oldhamii. https://cites.org/eng/node/21071.

[ref-12] Congdon JD, Dunham AE, Van Loben Sels R (1993). Delayed sexual maturity and demographics of Blanding’s turtles (*Emydoidea blandingii*): implications for conservation and management of long-lived organisms. Conservation Biology.

[ref-13] Dodd K (1990). Effects of habitat fragmentation on a stream-dwelling species, the flattened musk turtle *Sternotherus depressus*. Biological Conservation.

[ref-14] Durkin L (2012). Home range size, movements and refuge use of the Southeast Asian leaf turtle (*Cyclemys oldhamii*) in Phnom Kulen National Park, Cambodia. Unpublished Bachelor of Science honors thesis.

[ref-15] Fordham DA, Georges A, Brook BW (2007). Indigenous harvest, exotic pig predation and local persistence of a long-lived vertebrate: managing a tropical freshwater turtle for sustainability and conservation. Journal of Applied Ecology.

[ref-16] Frazer NB, Gibbons JW, Greene JL (1990). Exploring Fabens’ growth interval model with data on a long-lived vertebrate, *Trachemys scripta* (Reptilia: Testudinata). Copeia.

[ref-17] Garber SD, Burger J (1995). A 20-yr study documenting the relationship between turtle decline and human recreation. Ecological Applications.

[ref-18] Gibbons JW, Lovich JE (1990). Sexual dimorphism in turtles with emphasis on the slider turtle (*Trachemys scripta*). Herpetological Monographs.

[ref-19] Hall R, Henry P, Bunck C (1999). Fifty-year trends in a box turtle population in Maryland. Biological Conservation.

[ref-20] Heppell SS (1998). Application of life-history theory and population model analysis to turtle conservation. Copeia.

[ref-21] Inger RF, Colwell RK (1977). Organization of contiguous communities of amphibians and reptiles in Thailand. Ecological Monographs.

[ref-22] IUCN (2017). Thai Buddhist Council announces ban on ceremonial bird trade. https://www.iucn.org/news/thailand/201707/thai-buddhist-council-announces-ban-ceremonial-bird-trade.

[ref-23] IUCN (2018). IUCN Red List of Threatened Species. http://www.iucnredlist.org.

[ref-24] Ivan JS, White GC, Shenk TM (2013). Using simulation to compare methods for estimating density from capture—recapture data. Ecology.

[ref-25] Iverson J (1991). Life history and demography of the yellow mud turtle, *Kinosternon flavescens*. Herpetologica.

[ref-26] Kharel M, Chhetry DT (2012). Turtles of Kankai (Mai) river and their ethno-medicinal uses. Nepalese Journal of Biosciences.

[ref-27] Litzgus JD, Brooks RJ (1998). Testing the validity of counts of plastral scute rings in spotted turtles, *Clemmys guttata*. Copeia.

[ref-28] Litzgus JD, Mousseau TA (2006). Geographic variation in reproduction in a freshwater turtle (*Clemmys guttata*). Herpetologica.

[ref-29] Lyons JA, Natusch DJD, Shepherd CR (2013). The harvest of freshwater turtles (Chelidae) from Papua, Indonesia, for the international pet trade. Oryx.

[ref-30] R Development Core Team (2014). http://www.R-project.org.

[ref-31] Rai K (2004). Ecological distribution of *Cyclemys oldhamii* (Gray 1863) from Nepal. Our Nature.

[ref-32] Ramsay NF, Ng PKA, O’Riordan RM, Chou LM, Gherardi F (2007). The red-eared slider (*Trachemys scripta elegans*) in Asia: a review. Biological invaders in inland waters: profiles, distribution, and threats.

[ref-33] Reeves DJ, Litzgus JD (2008). Demography of an island population of spotted turtles (*Clemmys guttata*) at the species’ northern range limit. Northeastern Naturalist.

[ref-34] Rhodin A, Iverson J, Bour R, Fritz U, Georges A, Shaffer HB, Van Dijk P (2017). Turtles of the world annotated checklist and atlas of taxonomy, synonymy, distribution, and conservation status.

[ref-35] Seburn DC (2003). Population structure, growth, and age estimation of spotted turtles, *Clemmys guttata*, near their northern limit: an 18-year follow-up. The Canadian Field-Naturalist.

[ref-36] Severinghaus LL, Chi L (1999). Prayer animal release in Taiwan. Biological Conservation.

[ref-37] Shen J, Pike D, Du W (2010). Movements and microhabitat use of translocated big-headed turtles (*Platysternon megacephalum*) in southern China. Chelonian Conservation and Biology.

[ref-38] Shine R, Iverson J (1995). Patterns of survival, growth and maturation in turtles. Oikos.

[ref-39] Shoemaker KT, Breisch AR, Jaycox JW, Gibbs JP (2013). Reexamining the minimum viable population concept for long-lived species. Conservation Biology.

[ref-40] Sirois A, Gibbs J, Whitlock A, Erb L (2014). Effects of habitat alterations on bog turtles (*Glyptemys muhlenbergii)*: a comparison of two populations. Journal of Herpetology.

[ref-41] Smith GR, Iverson JB (2002). Sex ratio of common musk turtles (*Sternotherus odoratus*) in a north-central Indiana lake: a long-term study. The American Midland Naturalist.

[ref-42] Sodhi NS, Koh LP, Brook BW, Ng PKL (2004). Southeast Asian biodiversity: an impending disaster. Trends in Ecology & Evolution.

[ref-43] Stuart BL, Platt SG (2004). Recent records of turtles and tortoises from Laos, Cambodia, and Vietnam. Asiatic Herpetological Research.

[ref-44] Stuart BL, Rowley JJ, Neang T, Emmett DA, Som S (2010). Significant new records of amphibians and reptiles from Virachey National Park, northeastern Cambodia. Cambodian Journal of Natural History.

[ref-45] Sung Y-H, Hau BC, Karraker NE (2014). Reproduction of endangered Big-headed Turtle, *Platysternon megacephalum* (Reptilia: Testudines: Platysternidae). Acta Herpetologica.

[ref-46] Sung Y-H, Karraker NE, Hau BCH (2013). Demographic evidence of illegal turtle trapping on an endangered Asian turtle, *Platysternon megacephalum*. Conservation Biology.

[ref-47] Vamberger M, Durkin L, Kim C, Handschuh M, Seng R, Fritz U (2017). The leaf turtle population of Phnom Kulen National Park (northwestern Cambodia) has genetic and morphological signatures of hybridization. Journal of Zoological Systematics and Evolutionary Research.

[ref-48] Van Dijk P, Stuart B, Rhodin A (2000). Asian Turtle Trade.

